# The use of a *Psoroptes ovis *serodiagnostic test for the analysis of a natural outbreak of sheep scab

**DOI:** 10.1186/1756-3305-5-7

**Published:** 2012-01-10

**Authors:** Stewart TG Burgess, Giles Innocent, Francesca Nunn, David Frew, Fiona Kenyon, Alasdair J Nisbet, John F Huntley

**Affiliations:** 1 Moredun Research Institute, Pentlands Science Park, Bush Loan, Edinburgh. Midlothian. EH26 0PZ. Scotland. UK; 2 Biomathematics & Statistics Scotland, JCMB, King's Buildings, Edinburgh, EH9 3JZ. Scotland. UK

**Keywords:** Ectoparasite, Diagnostic, ELISA, *Psoroptes ovis*, Sheep scab

## Abstract

**Background:**

Sheep scab is a highly contagious disease of sheep caused by the ectoparasitic mite *Psoroptes ovis*. The disease is endemic in the UK and has significant economic impact through its effects on performance and welfare. Diagnosis of sheep scab is achieved through observation of clinical signs e.g. itching, pruritis and wool loss and ultimately through the detection of mites in skin scrapings. Early stages of infestation are often difficult to diagnose and sub-clinical animals can be a major factor in disease spread. The development of a diagnostic assay would enable farmers and veterinarians to detect disease at an early stage, reducing the risk of developing clinical disease and limiting spread.

**Methods:**

Serum samples were obtained from an outbreak of sheep scab within an experimental flock (n = 480 (3 samples each from 160 sheep)) allowing the assessment, by ELISA of sheep scab specific antibody prior to infestation, mid-outbreak (combined with clinical assessment) and post-treatment.

**Results:**

Analysis of pre-infestation samples demonstrated low levels of potential false positives (3.8%). Of the 27 animals with clinical or behavioural signs of disease 25 tested positive at the mid-outbreak sampling period, however, the remaining 2 sheep tested positive at the subsequent sampling period. Clinical assessment revealed the absence of clinical or behavioural signs of disease in 132 sheep, whilst analysis of mid-outbreak samples showed that 105 of these clinically negative animals were serologically positive, representing potential sub-clinical infestations.

**Conclusions:**

This study demonstrates that this ELISA test can effectively diagnose sheep scab in a natural outbreak of disease, and more importantly, highlights its ability to detect sub-clinically infested animals. This ELISA, employing a single recombinant antigen, represents a major step forward in the diagnosis of sheep scab and may prove to be critical in any future control program.

## Background

Sheep scab is caused by the mite *Psoroptes ovis *and is, arguably, the most important ectoparasitic disease of sheep in the UK. Recent data relating to the financial impact of sheep scab suggest that the disease costs the UK sheep industry in excess of £8 million per annum, including costs associated with lost performance, preventative measures, and treatment of affected animals; with the major costs relating to disease prevention [1]. Since its deregulation as a notifiable disease in 1992, sheep scab has become endemic throughout the UK [2]. The disease is highly contagious, causing considerable pruritis and irritation and is a major welfare concern [3]. Current disease control strategies are reliant upon chemotherapy; however, concerns over residues, eco-toxicity and the development of acaricide resistance have led to questions being raised regarding the sustainability of current strategies and an interest in the development of alternative control methods [4,5]. The chemicals used to treat sheep scab are also relied upon for control of gastrointestinal (GI) parasites, as such limiting their use in sheep scab control is essential to reduce development of resistance in both mites and GI parasites, prolonging drug efficacy for these economically important diseases [6]. Strategies designed to control the spread of sheep scab are reliant upon the availability of a diagnostic assay capable of accurately detecting infested animals, thus enabling effective quarantine and treatment. A major problem in disease control is the rapid spread of infestation, normally via direct contact or by transfer of mites from infested fleece, fence posts, farm or veterinary machinery and workers [3,7]. During the early stages infestations are not obvious and animals often appear clinically normal [7,8]. This subclinical stage can last for several weeks during which animals can act as a source of mites [7,8]. At present, diagnosis of sheep scab is based upon animal history, clinical signs and confirmation through identification of *P. ovis *mites in scrapings from lesional skin [8]. Inevitably, animals with sub-clinical infestations or minimal lesions will evade detection. For control or eradication programs to be successful it is crucial that all infested animals are identified, including subclinical cases.

Targeted treatments of *P. ovis*-infested sheep, based on flock serology, have been used successfully to reduce the incidence of sheep scab [9,10]. Our group has recently developed a novel diagnostic enzyme linked immunosorbent assay (ELISA) capable of accurately detecting *P. ovis *infestation in sheep [11]. Unlike previous tests, this assay is based on detection of host antibodies specific to a recombinant mite allergen, termed Pso o 2 (rPso o 2) and in our hands, has proven to be sufficiently sensitive to detect sheep scab infestation within 2-3 weeks of contact. This paper describes the first use of this diagnostic assay for the analysis of a natural outbreak of sheep scab that occurred during 2009 within an experimental flock at the Moredun Research Institute, Edinburgh. The study involved the collection of sera from animals at three dates; these coincidentally occurred prior to the outbreak of sheep scab, shortly after clinical signs of sheep scab appeared in the flock and finally after the flock was treated. Fortuitously this allowed analysis of antibody levels in individual animals over the course of sheep scab infestation and also enabled association of antibody levels with observed clinical signs. This analysis has further demonstrated the ability of this diagnostic assay to accurately detect sheep scab infestation in a natural outbreak and, more importantly, highlights the potential of the assay to detect sheep scab prior to the appearance of clinical signs.

## Methods

### Experimental animals

The study described herein was designed to assess differences between treatment regimes for natural infections of GI parasites of sheep and was not designed for the analysis of a sheep scab outbreak. Ethical approval for the study was obtained from the Moredun Research Institute Experiments Committee. All serum samples described in this study were taken from the original trial. Archived samples were made available to the authors for analysis once it was apparent that sheep scab was present within the group, as such no further ethical approval was required or sought. 160 twin Texel-cross lambs reared at the Moredun Research Institute, Edinburgh were separated along with their Ewes into eight individually fenced one acre paddocks (Paddocks 1-8), with 20 lambs per paddock (12/05/09). Each paddock (1-8) was separated by single fencing and isolated from the outside by double fencing, or single fencing and an empty paddock. Animals were handled within an exclusive on-site facility adjacent to paddock 2 and processed in paddock order starting with paddock 1 and ending with paddock 8. Blood samples were taken at three dates; pre-outbreak (23/06/2009), mid-outbreak (31/08/2009) and post-treatment (13/10/2009). In addition, later stage serum samples were obtained from 33 randomly selected animals from the same trial on the 13/05/2010 to assess the longevity of the antibody response.

Sheep scab was diagnosed on a separate area of the farm (16/06/09) however, the study animals were unaffected at this time. Ewes were briefly removed from their lambs to be clipped; this was carried out in a shared facility on the Institute farm (07/07/2009) and ewes were then moved back to their respective paddocks. Shortly afterwards the lambs were weaned (14/07/2009) and ewes moved to fresh pasture. Sheep scab was subsequently observed in the study animals (initially in Paddock 5) (24/08/09). All sheep were assessed by visual inspection of the fleece and skin for clinical signs of sheep scab (31/08/09). The following parameters were assessed; 1) physical, i.e. obvious presence of mites, staining or removal of fleece and evidence of skin lesions; 2) behavioural, i.e. signs of discomfort or excessive itching and an itch response (lip smacking) following contact. Based on these parameters, each animal was assigned a clinical score from 0-5, depending on the severity of clinical signs. Mild physical signs were classified as staining or removal of fleece but with no obvious lesions or presence of mites. Moderate physical signs as staining or removal of fleece combined with the presence of skin lesions. Severe physical signs were defined as staining and removal of fleece, obvious skin lesions and presence of mites. Mild behavioural signs were defined as excessive itching and discomfort. Moderate behavioural signs as excessive itching and discomfort combined with a mild itch response following contact with lesional skin. Severe behavioural signs as excessive itching and discomfort combined with a strong itch response following contact with lesional skin. All sheep in the study were dipped with the organophosphate Diazinon (02/09/2009) to treat for sheep scab, with the treatment being repeated after clinical signs persisted in some animals (30/09/2009).

### Sample handling and preparation

Whole blood samples were collected into vacutainer tubes without anti-coagulant and clotted overnight at +4°C. Samples were centrifuged at 5,000 *g *for 5 minutes at room temperature, serum removed by decanting and stored at -20°C.

### ELISA sample processing

Recombinant Pso o 2 (rPso o 2) antigen was produced for the ELISA as previously described [11] and samples were analysed in duplicate with the sheep scab diagnostic ELISA following a standard operating procedure as previously described [11]. Plates were read at 450 nm on a microplate reader (Tecan, UK) and well OD values were obtained. Positive controls consisted of pooled hyper-immune sera (animals experimentally infested with *P. ovis *for a second time, following a primary infestation and successful treatment) diluted 1 in 200 to give an OD_450 _of 0.85 (± 20%), and this sample was included on each plate as an interplate control. Plate readings were normalised to that OD_450 _reading and any control sample outside the range ± 20% of OD_450 _0.85 led to the plate being repeated. Sample test readings were averaged, normalised to the control and the average background reading for each plate subtracted from the test readings to produce an adjusted/corrected sample reading.

### Data analysis - interpretation

We previously described the analysis of 433 *P. ovis*-negative and 58 *P. ovis*-positive samples using this ELISA [11]. The data were analysed using receiver operator characteristic (ROC) curves [12,13] in order to determine an OD_450 _threshold above which a sample from a sheep with unknown disease status could be classified as positive for *P. ovis *infestation [11]. Using these reference data, it was concluded that samples should be considered positive for sheep scab if the adjusted sample test OD_450 _reading was > 0.06 absorbance units (AU). Using this threshold the assay provided a test sensitivity of 0.93 and a specificity of 0.9. Following this regime, serum samples taken from each animal were classified as being either positive or negative for scab. The classification scheme used to define TP, FP, TN and FN samples was as follows: TP = Animal with disease correctly classified as positive; TN = Animal without disease correctly classified as negative; FP = Animal without disease incorrectly classified as positive; FN = Animal with disease incorrectly classified as negative.

### Statistical analysis

All data analyses were conducted in R [14]. A general linear model was used to analyse log (ELISA OD_450_) + 0.005 between paddocks over the three sample times. This was deemed appropriate as the errors from the model were normally distributed. Comparison of the number of animals over the cut-off to the number under the cut-off was analysed using a generalised linear binomial model with logistic link. Non-parametric techniques were used to compare groups of ELISA OD_450 _values otherwise. These included Kruskal-Wallace analysis, where multiple groups were compared (e.g. comparing paddocks or clinical indices), and Mann-Whitney U test, where there were two groups (e.g. clinical vs non-clinical). Table 4 was generated following analysis of the data using a binomial generalised linear model, which demonstrated that the best model was one with paddock, time-point and an interaction between the two. However, due to the sparsity of the contingency table, many standard errors of the parameters were inappropriately high. Therefore, a bespoke function was written to calculate the likelihood for the model. This was then maximised to determine the maximum likelihood estimators. The values for each parameter which increased the deviance (-2*log-likelihood) by 3.84 (5% critical value of the Chisquare distribution with 1 degree of freedom) represent the limits of the 95% confidence interval for that parameter. To ascertain whether there was any difference between ELISA values in clinical and non-clinically affected animals it is not sufficient to demonstrate that there is no significant difference between groups, as this could be a result of low animal numbers and a resulting lack of power. Formally, the statistical test needs to be formulated as an equivalence problem. The Mann-Whitney U statistic can be used to determine the area under the receiver operating characteristic (ROC) curve (the AUC) [15]. If a test is perfectly unable to distinguish between two groups then the theoretical value of the AUC is precisely 0.5 [15]. We assumed that a test cannot distinguish between groups if this value lies between 0.3 and 0.7 (the ROC curve being symmetric if we invert the test).

## Results

### Clinical signs of disease

Twenty seven of the 160 animals presented with clinical signs of sheep scab infestation, ranging from very mild to severe (Table 1). Twelve animals showed behavioural signs of infestation but no obvious physical signs (Clinical score = 1). Six animals showed mild physical signs but no obvious behavioural signs (Clinical score = 2). A single animal in paddock 5 presented with mild physical and behavioural signs (Clinical score = 3), whilst a further 4 animals showed moderate physical and behavioural signs (Clinical score = 4). Four animals showed severe physical and behavioural signs (Clinical score = 5) and one animal died of an unrelated cause prior to the physical examination. The remaining 132 animals showed no obvious physical or behavioural signs (Clinical score = 0).

**Table 1:** Clinical scores mid-outbreak

Clinical Score	Number of animals	Paddock Number
**0**	132^#^	All

**1**	12	2, 6*, 7, 8

**2**	6	5, 7

**3**	1	5

**4**	4	5, 6

**5**	4	5

Distribution of clinical scores following inspection of animals at the mid-outbreak sampling. 0 = No obvious physical or behavioural signs; 1 = Mild behavioural but no obvious physical signs; 2 = Mild physical signs but no obvious behavioural signs; 3 = Mild physical signs combined with mild behavioural signs; 4 = Moderate physical signs combined with moderate behavioural signs; 5 = Obvious and severe physical signs combined with severe behavioural signs. *One serum sample unavailable at the mid-outbreak sampling. ^#^One animal died of an unrelated cause.

### Comparison of ELISA results with clinical signs

At the mid-outbreak sampling point 27 animals showed varying degrees of clinical signs. None had pre-outbreak OD_450 _ELISA values > 0.06, indicating that they were negative pre-outbreak. At the same sampling point 25 of these clinically positive animals had OD_450 _values > 0.06, one was below this cut-off, and one sample was unobtainable. The two animals without positive ELISA readings at the mid-outbreak sampling period had OD_450 _values above the threshold at the subsequent sampling, indicating either very early infestation or a delayed reaction. Of the 130 animals for which OD_450 _values were available pre-outbreak, 124 had OD_450 _values < 0.06 and six > 0.06: thus representing potential false positives (FPs). At the mid-outbreak sampling 105 of the 132 clinically negative animals had OD_450 _values > 0.06, 23 of these were below the cut-off, and 4 samples were unobtainable. At the post-treatment sample the OD_450 _values of 70 of the 132 samples were > 0.06, 59 were below the cut-off and 3 were unobtainable. The clinically negative, test positive group will include both FP and sub-clinically affected animals.

The specificity of the test has previously been estimated at 0.90 [11]. Therefore, if the 23 (27 if we include the 4 unobtainable results) clinically negative, test negative animals represent 90% of all truly negative animals then we would expect 2.6 (3) FPs. We can therefore infer that the majority of the 105 test-positive, clinically negative animals represent sub-clinical infestations. To compare the performance of the test in clinical and sub-clinical animals we can compare the distribution of OD_450 _values from all clinically positive animals with those which were clinically negative but test positive. Since we believe that the majority of the latter group represent sub-clinical cases, any bias due to misclassification should be small.

There was no statistically significant difference between the median OD_450 _values of non-clinical, above-threshold animals and animals with each of the five levels of clinical signs recorded (Kruskal-Wallace chi-squared = 7.20, p = 0.21). Due to the low numbers of animals with clinical signs these animals were combined into a single group and again no significant difference in average location statistic was observed between these and the non-clinical, above threshold animals (Mann-Whitney U = 1166, p = 0.39). To check the sensitivity of these results to the choice of cut-off, the analysis was repeated using OD_450 _cut-offs of 0.04 (Mann-Whitney U = 1275, p = 0.27) and 0.10 (Mann-Whitney U = 309.5, p-value = 0.20). Using the pre-defined OD_450 _cut-off of 0.06 to define sub-clinical cases, the estimated AUC for the ability of the test to discriminate between clinical cases and non-clinical, above threshold animals was 0.44 with a 95% confidence interval (0.32, 0.57). We can therefore conclude that there is no evidence for differences in the distribution of ELISA values in the clinical and non-clinical, above-threshold groups. We therefore infer that at this cut-off value the test can equally identify both clinical and sub-clinical cases.

Whilst these findings highlight the difficulty of diagnosing sheep scab through the presence of physical or behavioural signs alone, they may also provide a demonstration of the ability of the ELISA to diagnose sub-clinical infestation.

### False positive and false negative samples

ELISA results were used to determine the infestation status of all 160 animals throughout the course of the outbreak (Table 2). At the pre-outbreak sampling date 6 animals tested positive and 152 negative (2 samples were unavailable). At the mid-outbreak sampling date 120 animals tested positive and 34 negative (6 samples were unavailable). At the post-treatment sampling date 90 animals tested positive and 65 negative (5 samples were unavailable). Previous ROC curve analysis of ELISA test results from 491 clinically negative (n = 433) and positive (n = 58) samples showed that the AUC was 0.97, which equates to a test accuracy of 97%, indicating an excellent level of discrimination between clinical cases and *P. ovis-*free animals [11,15]. Using the OD_450 _threshold of > 0.06 AU the ELISA showed a test sensitivity of 93% and test specificity of 90% [11]. As such we would expect to be able to accurately define 90% of true negative (TN) samples and 93% of true positive (TP) samples, leaving 10% FP and 7% false negative (FN) samples.

**Table 2:** Distribution of animals classified as positive or negative by ELISA and mean antibody levels during outbreak of sheep scab

Sampling time	Positive	Negative	Other	Mean antibody level	Range	Standard error (SEM)
**Pre-outbreak**	6	152	2	0.01	0 - 0.11	0.0016

**Mid-outbreak**	120	34	6*	0.12	0.01 - 1.16	0.01

**Post-treatment**	90	65	5*	0.08	0 - 0.47	0.0056

Numbers of animals classified as positive or negative for sheep scab based on their ELISA results throughout the outbreak. Positive animals classified as having an OD_450 _value > 0.06. Also shown are the mean ELISA OD_450 _values, the range of values across all 160 animals throughout the outbreak of sheep scab and the standard error associated with each mean.*1 animal died of an unrelated cause, other samples unavailable due to unobtainable serum samples.

The identification of 6 potential FPs during this study (3.8%) was in fact significantly (p ≤ 0.009) lower than would be expected (10% or ~16 FPs) based on previous estimates of test properties [11] and hints at a previous under-estimation of the test specificity (here estimated to be 96.2% but previously 90%). FNs were more difficult to define. However, these can be classified as animals with clinical signs of disease but with a negative test result. In this case we observed 2 FNs that had clinical scores of 1 and 4 respectively during the mid-outbreak assessment and both had ELISA OD_450 _levels < 0.06 at this time point. However, both were then correctly classified as positive at the subsequent sampling period. This demonstrates a FN rate of 2/27 animals (7.4%) which is not out of line with the predicted 7% rate of FNs based on previous estimations (p = 0.94). It also demonstrates that test sensitivity strongly depends on the infestation history of the animals being tested, an aspect of the epidemiology which will be explored more thoroughly in the next section.

### Analysis of antibody levels over the course of infestation

**Figure 1:**
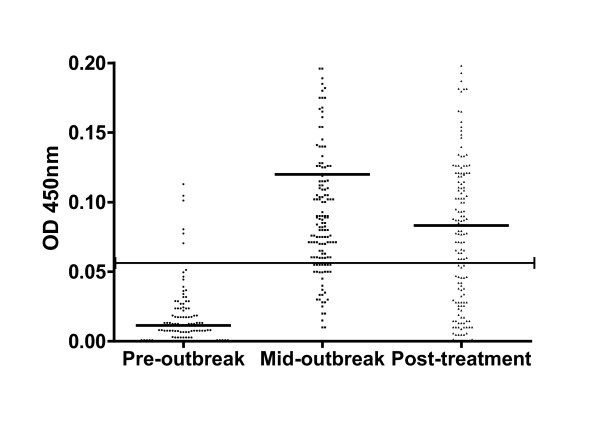
**Scatter plot of individual antibody levels during sheep scab outbreak**. Horizontal black line indicates the 0.06 OD_450 _threshold above which samples were classified as positive for sheep scab. Animals were treated after the mid-outbreak sampling point. Group means indicated by black horizontal lines within individual scatter plots. Thirty seven extreme values (OD _450 _values > 0.2) removed from mid-outbreak and post-treatment sample sets to aid with data visualisation, though still contributed to summary statistics.

The antibody levels for all animals in the study at each time point are summarised in Table 2 and presented in Figure 1. There were significant differences in the mean ELISA OD_450 _levels pre-outbreak *vs*. mid-outbreak, pre-outbreak *vs*. post-treatment and mid-outbreak *vs*. post-treatment (p ≤0.0001). Low numbers of potential FPs (n = 6) were observed at the pre-outbreak sampling period (mean OD_450 _= 0.01) whilst all clinically positive animals were successfully classified by their mid-outbreak or post-treatment sample along with a number of sub-clinical cases (mean OD_450 _= 0.12). From the data presented in Figure 1 and Table 2 it can be seen that post-treatment the overall mean OD_450 _dropped significantly to 0.08 (p ≤ 0.001), highlighting the reduction in antibody levels across the flock within 6 weeks post-treatment.

### Paddock data analysis

**Figure 2:**
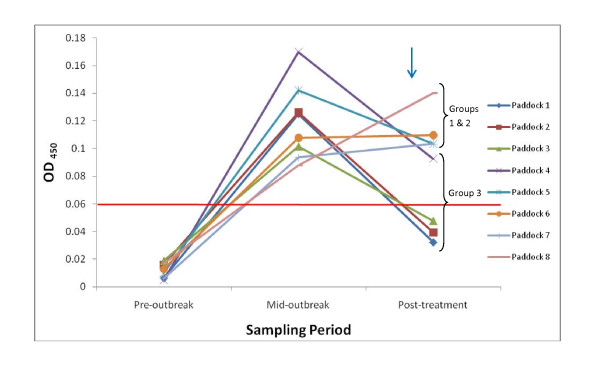
**Mean antibody levels across all paddocks throughout the outbreak**. Vertical blue arrow indicates treatment. Horizontal red line indicates 0.06 OD_450 _cut-off. Groups 1 and 2 (mean positive for sheep scab post-treatment) covers paddocks 5-8 and Group 3 (mean negative post-treatment) 1-4.

Fourteen animals remained negative throughout the study with ELISA results below the OD_450 _threshold of > 0.06 (mean OD_450 _= 0.023, range = 0.00 - 0.06) and showing no clinical signs. These animals were spread throughout paddocks (p) 1-6 (p1 n = 3, p2 n = 3, p3 n = 4, p4 n = 2, p5 n = 1, p6 n = 1) with no negative animals observed in paddocks 7 and 8. A single animal was classified positive throughout the study (paddock 8); in fact, its serum antibody levels increased consistently throughout the study, peaking post-treatment indicating that this animal may have been a FP even at the pre-outbreak sampling date. As would be expected the majority (141 of 160 or 88%) of animals were classified as positive at either the mid-outbreak, or the post-treatment sampling period. A majority of these animals can be classified into one of three groups: Group 1 = negative pre-outbreak and positive at the remaining two sampling dates (n = 68); Group 2 = negative pre- and mid-outbreak but positive post-treatment (n = 15); Group 3 = negative pre-outbreak, positive mid-outbreak and negative again post-treatment (n = 45). Two interesting patterns were observed with these animals (Figure 2), Groups 1 and 2 were spatially distinct with the majority of these animals located in paddocks 4-8 (p1 n = 1, p2 n = 4, p3 n = 3, p4 n = 13, p5 n = 14, p6 n = 15, p7 n = 19, p8 n = 14). Group 3 formed a spatial complement, with the majority of animals located in paddocks 1-5 (p1 n = 15, p2 n = 11, p3 n = 9, p4 n = 5, p5 n = 4, p6 n = 1). The remaining animals which tested positive by ELISA during the outbreak belonged to one of 4 additional groups all of which were small in number and a number of which probably represented FP animals: i) positive pre- and mid-outbreak, and negative post-treatment (n = 2, paddocks 2 and 3); ii) positive pre-outbreak and negative for the remaining samples (n = 1, paddock 3); iii) positive pre-outbreak and post-treatment but negative mid-outbreak (n = 2, paddocks 6 and 8); or iv) animals for which samples were unavailable at one or more time points (n = 12).

To identify effects attributable to individual paddocks we applied a binomial test model to the longitudinal data using the optimum test cut-off of an OD_450 _> 0.06, with all results being classified as either test positive or negative. This analysis demonstrated that no paddocks were significantly different from the overall average during the mid-outbreak sampling period, although paddock 8 was at marginally significantly lower probability of demonstrating positive tests (p < 0.1) (Table 3). However, post-treatment, paddocks 1-3 had statistically significantly (p < 0.05) lower probabilities of demonstrating positive results, while paddocks 7 and 8 showed statistically significantly higher levels (p < 0.05) of test positivity than the mid-outbreak average (Table 3). These results point to the presence of a spatial discrimination between the paddocks, with paddocks 1-3 showing a more rapid or stronger recovery post-treatment than paddocks 7 and 8. The reasons behind this paddock effect are unclear, however, may be due to the way in which the disease spread within and between individual paddocks, or be influenced by the order in which the animals within each paddock were handled and treated (See Discussion).

**Table 3:** Data from the fitting of a binomial model to longitudinal data

Paddock	Sampling Period	RR	95% CI
**1**	Mid-outbreak	1.07	0.86-1.16

**2**	Mid-outbreak	1.12	0.93-1.18

**3**	Mid-outbreak	0.95	0.70-1.10

**4**	Mid-outbreak	0.89	0.64-1.06

**5**	Mid-outbreak	1.07	0.86-1.16

**6**	Mid-outbreak	1.06	0.84-1.16

**7**	Mid-outbreak	0.93	0.68-1.10

**8**	Mid-outbreak	*0.91*	*0.63-1.09*

**1**	Post-treatment	**0.063**	**0.0047-0.25**

**2**	Post-treatment	**0.24**	**0.079-0.48**

**3**	Post-treatment	**0.18**	**0.047-0.41**

**4**	Post-treatment	0.83	0.57-1.03

**5**	Post-treatment	1.00	0.76-1.13

**6**	Post-treatment	1.12	0.93-1.18

**7**	Post-treatment	**1.18**	**1.08-1.18**

**8**	Post-treatment	**1.18**	**1.06-1.18**

All Relative Risk (RR) are relative to the mean risk at the mid-outbreak sampling period. Estimates with statistically significant (p < 0.05) p-values shown in bold type. Estimates with marginally significant (p < 0.1) p-value in italics.

### Antibody longevity analysis

To assess the longevity of the antibody responses to *P. ovis *Pso o 2 antigen, samples were obtained from 33 animals from the same trial nine months after the sheep scab outbreak was noticed and eight months post-treatment. All animals tested had previously been classified as positive for sheep scab from the ELISA results of either their mid-outbreak or post-treatment samples. Twenty seven animals (81%) were negative for sheep scab eight months post-treatment, with levels of circulating antibody that would be considered negative, using the proposed cut-off value (mean OD_450 _= 0.02 (range = 0 - 0.06)). Six of the 33 animals remained positive for sheep scab (19%) with low or border line OD_450 _values (mean = 0.076 (range = 0.07 - 0.1), and the OD_450 _values for 4 of these 6 animals were in fact lower than their respective OD_450 _values taken 2 weeks post-treatment (13/10/2009), indicating a slow decrease in the antibody response. These results are consistent with the level of circulating antibody observed in infested animals being transient, declining following treatment.

## Discussion

Here we have shown that this diagnostic assay for the serological detection of sheep scab infestation [11] is able to effectively detect disease during a natural outbreak. This analysis has also demonstrated the ability of the assay to detect sub-clinical disease and has shown that the level of circulating antibody diminishes with time after successful treatment, both of which are crucial attributes during any future control program.

### False positive and false negative samples

The threshold of > 0.06 AU for ELISA readings (OD_450_) delivers a test sensitivity of 0.93 and specificity of 0.9 [11]. At this threshold the ELISA identified 6 potential FPs (3.8%, positive for sheep scab pre-outbreak) and 2 potential FNs (7.4%, clinical signs of disease but with a negative ELISA result). Although the number of FNs was in line with previous understanding of the test properties, suggesting that the sensitivity in the field is equivalent to that estimated previously, the level of FPs was significantly lower than expected and may indicate a higher overall test specificity (96.2%); where this has previously been estimated as 90% [11]. This may indicate that samples used in the calculation of the original OD_450 _threshold were not representative of the population of negative animals. This is borne out by a result in the original paper which compared a further 20 negative animals and found them to have significantly lower OD_450 _values.

Altering the threshold above which we classify a sample as positive will also change the test sensitivity and specificity and therefore alter the number of FP and FN results [16]. For example, increasing the threshold OD_450 _to 0.1 absorbance units would provide predicted test sensitivity and specificity of 79.3% and 99.1%, respectively, thus biasing the test towards increased specificity and therefore reducing the number of FPs, whilst increasing the number of FNs. In contrast, reducing the threshold would have the opposite effect, biasing the test towards increased sensitivity at the expense of specificity, increasing the number of FPs whilst reducing the number of FNs. Thus, the selection of a test threshold is very much a balance between specificity and sensitivity for optimal test performance [16-18]. For sheep scab, an ideal test would aim to reduce the rate of FPs as these could lead to unnecessary quarantine and/or treatment of non-infested flocks with the financial implications of unnecessary treatment. Biasing the assay towards the reduction of FPs leads to a moderate increase in the number of FNs, and with this type of diagnostic assay FNs are unavoidable. However, this test is expected to be used on a number of animals simultaneously that represent either a whole flock, or a group of co-housed animals. If any animal is positive then the whole group would be treated. Due to the rapid spread, it is unlikely that only a single animal in a group of sheep would be infested, so, even with two truly positive animals among the tested animals the probability that the group is missed and therefore not treated is reduced to 7.4% in the above example (group sensitivity of 92.6%). Further cost modeling would allow the determination of the optimum cut-off depending on the presence or absence of clinical signs and management of the animals, i.e. hill *vs*. lowland flocks, where increased density of lowland farming could lead to a more rapid spread of infestation. Although the results shown here suggest greater test sensitivity and specificity than previously thought, the ideal threshold may be slightly higher than the OD_450 _> 0.06 described herein - for example the cut-off of 0.1 discussed above would reduce the level of FPs to less than 1% whilst increasing the level of FNs to approximately 10%, which may represent a good compromise.

### Comparison of ELISA results with clinical signs

We have demonstrated that there is no evidence that the ELISA values for clinical and non-clinical, above threshold animals come from anything other than indistinguishable distributions using this test. Since most of the non-clinical, above threshold animals are likely to represent sub-clinical cases this indicates that the test is equally able to detect sub-clinical as clinical cases if their OD_450 _value is above threshold. It is likely that the response measured by the ELISA is an early response to the presence of the mites, and not affected by the degree of damage caused by the mites or self-trauma. This is crucial for any *Psoroptes*-diagnostic assay as the success of any disease control or eradication program is entirely dependent on the availability of a diagnostic assay sensitive enough to identify diseased animals prior to the advent of clinical signs. This is important for sheep scab, as animals with obvious physical and behavioural signs are relatively straightforward for a trained veterinarian or experienced farmer to diagnose but sub-clinical animals provide a far greater challenge to disease control. However, it should be noted that we cannot unequivocally state that the sub-clinical animals detected in this study would have gone on to develop clinical disease due to the ethical and legal duty to treat all suspected animals. The fact that all but five of these potentially sub-clinical animals were serologically negative pre-infestation, along with the low levels of cross-reactivity detected with other parasitic diseases of sheep including gastrointestinal nematodes, keds and lice, adds further support to these findings [11]. The fact that these sheep were maintained in paddocks with known infested animals points to an obvious source of infestation and this is further supported by a previous study which demonstrated the active spread of sheep scab in a matter of weeks across a flock of sheep following the introduction of single infested animals [19].

### Analysis of antibody levels over the course of infestation

The presence of highly statistically significant (p ≤0.001) differences between the average antibody levels seen in pre-outbreak, mid-outbreak and post-treatment sampling periods in this study demonstrates that the ELISA was capable of both accurately detecting sheep scab and of reflecting changes in disease following treatment. In conjunction with our previous analysis [11] this offers further validated support for the use of this test in any future sheep scab control or eradication program.

### Paddock data analysis

The outbreak was first noticed with the appearance of clinical signs within paddocks 5 and 6 and the majority of animals (19/27) showing obvious clinical signs mid-outbreak were located within these paddocks. The remaining clinical animals were spread across the paddocks. Paddocks 5 and 6 had more noticeable infestations than the other paddocks and this was expressed in both the ELISA data and clinical scores offering further support for the hypothesis that the outbreak may have started in Paddock 5. All animals were processed in a specific order, once every two weeks, starting with paddock 1 and ending with paddock 8, with one paddock being processed at a time before being returned to their original paddock. *P. ovis *mites are able to survive off host for up to 38 days, but may only remain infective for 15-16 days [20,21]. It is therefore unlikely that mites would have been transferred from paddock 5 to animals in paddocks 1-4 as two weeks would pass before these animals came back through the shared handling facility. Conversely, animals in paddocks 6-8 were processed directly after the animals in paddock 5. Therefore if we assume that the infestation began in paddock 5, then paddocks 6-8 would have had an increased risk of mite exposure compared to animals in paddocks 1-4. It should also be noted that individual paddocks were separated by single fencing: as such, transfer of mites between adjacent paddocks cannot be ruled out, but would at least be limited. This hypothesis is supported by the ELISA data, with paddocks 7-8 showing statistically significantly higher mean levels of test positivity and paddocks 1-3 showing statistically significantly lower mean test positivity post-treatment (Table 3). Another hypothesis relates to the dipping regime applied to these animals; again this was performed in paddock order. Dipping was performed using an automated dipping apparatus processing one paddock (20 animals) at a time, with fresh dip being added after approximately 30 animals. However, post-treatment, the animals in paddocks 6-8 continued to show clinical signs, and as such, all animals in the study were dipped for a second time. It is possible that the animals in paddocks 6-8 did not get sufficient levels of acaricide to effectively kill all mites upon first treatment. This could be due to depletion or fouling of the chemicals in the dip during the dipping of the earlier paddocks. However, this cannot be confirmed and the dip was regularly topped up, although it could explain the need for an additional treatment in these paddocks and the significantly higher levels of test positivity observed. Unfortunately it is impossible to confirm either of these hypotheses although they may partly explain why the ELISA results for animals in paddocks 5-8 appeared to decline more slowly than those in paddocks 1-4 following treatment.

Fourteen animals were found to be negative throughout the study and these were spread across paddocks 1-6. Interestingly, paddocks 5 and 6 had only one negative animal each, with no negative animals being found in paddocks 7 and 8 and this may offer further support for the explanation described above.

### Antibody longevity analysis

An important feature of a good diagnostic assay is the ability to discriminate between animals with disease and those that were previously infested but are now disease-free. One of the drawbacks of antibody-based diagnostic assays is the persistence of the antibodies in the circulation, which can lead to FP results following treatment. Encouragingly, the data presented here show that the level of circulating antibodies with specificity to Pso o 2 reduced with time with only borderline responses detectable in a few of the tested animals (19%) 8 months post-treatment. This is consistent with previous studies which have shown that *P. ovis*-specific antibodies may persist in the circulation 3-9 months after treatment (Peter Bates, Personal Communication). These findings show that the circulating antibodies are transient, declining following treatment and indicating that their impact on the level of FPs is likely to be minimal.

### Source of sheep scab outbreak

It is not possible to definitively identify the source of the sheep scab outbreak within this experimental flock. However, it is highly likely that sheep scab was introduced into paddock 5 when the ewes were clipped at the farm's shared handling facility. At this point animals were moved from a very low risk area of effective quarantine into a shared facility which posed a much higher risk of infestation and it was at this stage that the chances of exposure from the outbreak prevalent in other parts of the farm were at their greatest.

## Conclusions

The occurrence of a natural outbreak of sheep scab within an experimental flock enabled the analysis of the performance of a diagnostic ELISA in an environment where samples were available from all animals pre-infestation, mid-outbreak and post-treatment. This situation facilitated the validation of the ELISA, further demonstrating its ability to detect disease in a natural outbreak, not only in animals with obvious clinical signs but more importantly in animals showing no physical or behavioural signs, but that had been 'in contact' with infested animals. This latter sub-clinical group are far more important from a disease control perspective, because if undetected they may act as a source of infestation exacerbating disease spread. The ELISA described herein employing a single recombinant protein represents a major step forward for the diagnosis of sheep scab and may be critical in any future control program.

## Competing interests

The authors declare that they have no competing interests.

## Authors' contributions

STGB conceived and designed the study, performed clinical assessments, contributed to data analysis and wrote the manuscript. GI performed the statistical analysis and helped to prepare the manuscript. FN prepared the samples and performed ELISAs. DF helped with the clinical assessments and sample preparation. FK designed the study, helped with the clinical assessments, sample collection and preparation of the manuscript. AJN helped with the study design and statistical analysis and with the manuscript preparation. JFH conceived and designed the study and helped to prepare the manuscript. All authors have read and approved the manuscript.
